# Caste, wealth and geographic equity in program reach and expected outcomes: an exploratory analyses of *Suaahara* in Nepal

**DOI:** 10.3389/fnut.2024.1464902

**Published:** 2024-12-30

**Authors:** Kenda Cunningham, Ramesh P. Adhikari, Poonam Gupta, Shalini Suresh, Jiaxin Chen, Aman Sen, Manisha Laxmi Shrestha, Kristine Garn, Pooja Pandey Rana, Debendra Adhikari

**Affiliations:** ^1^Helen Keller International, New York, NY, United States; ^2^Faculty of Epidemiology and Population Health, London School of Hygiene and Tropical Medicine, London, United Kingdom; ^3^Helen Keller International, Kathmandu, Nepal; ^4^Income and Benefits Policy Center, The Urban Institute, Washington, DC, United States; ^5^Center for Human Nutrition, Department of International Health, Johns Hopkins Bloomberg School of Public Health, Baltimore, MD, United States; ^6^FHI360, Kathmandu, Nepal; ^7^United States Agency for International Development, Kathmandu, Nepal

**Keywords:** equity, Nepal, nutrition, implementation science, social and behavior change, caste, wealth, geography

## Abstract

**Introduction:**

Monitoring and evaluation of maternal and child nutrition programs typically concentrates on overall population-level results. There is limited understanding, however, of how intervention reach and expected outcomes differ among sub-populations, necessary insight for addressing inequalities. These analyses aim to determine if maternal exposure to social and behavior change (SBC) interventions is associated with scales of maternal practices (antenatal care, iron and folic acid in pregnancy, diet in pregnancy, postnatal care, iron and folic acid postpartum, and maternal dietary diversity) and child practices (institutional birth, health mothers’ group participation, growth monitoring and promotion, early initiation of breastfeeding and infant and young child feeding) in Nepal, overall and by wealth, caste, and geography.

**Methods:**

Cross-sectional data from 2022 from the USAID-funded Suaahara program in Nepal were used for analysis. The study focused on households with children aged 0–2 years (*N* = 1815). Descriptive analysis was followed by regression models, adjusted for potentially confounding child, mother, and household factors, as well as community-level clustering.

**Results:**

Greater intensity of maternal engagement with the SBC interventions was positively associated with both scales for maternal and child nutrition-related practices. The magnitude of the positive associations, however, was less for the socially excluded caste versus others for maternal nutrition; there was almost no caste-based variation in associations for child nutrition. Positive associations were found only among the lower 40% socio-economically when mothers engaged with at least two platforms for maternal nutrition and at least three platforms for child nutrition. In contrast, engagement with one platform for the relatively wealthier was positively associated with both maternal and child outcomes. Similarly, the positive associations were stronger among those residing in the terai (lowland plains) than those in the hills and mountains for both maternal and child outcomes.

**Discussion:**

The scope for improving maternal and child nutrition practices is significant, in part via well-designed, targeted, SBC programs. These analyses highlight the importance of considering different domains of equity both in implementation and related research. Further mixed methods research is needed to more deeply explore why certain types of interventions reach and/or have a greater effect on sub-populations.

## 1 Introduction

Investment in promoting healthy maternal and child nutrition practices in low- and middle-income countries is crucial to reduce morbidity and mortality as well as improve productivity, cognition, and executive function (1, 2). Improving maternal and child nutrition outcomes in South Asia remains a global priority. There is a wealth of literature that points to the importance of targeting interventions to mitigate the impacts of undernutrition on mothers and children during the thousand-day period between pregnancy and a child’s second birthday. Optimizing nutrition during this critical period can have long-lasting benefits for the child’s physical and mental development (3–5).

Despite progress made in the last few decades (6), undernutrition remains a severe public health problem for mothers and children. This global burden of undernutrition is not experienced equitably—every country in the world sees significant inequalities by factors such as location, remoteness, age, sex, education, and wealth. These health inequities are driven by social determinants of health including differences in access to and availability of quality health services, sufficient and healthy food, wealth, education, and social inclusion (7).

The Government of Nepal (GoN) has made significant strides in the past few decades to address poor maternal and child nutrition, building on its early success in increasing access to Vitamin A supplementation through the establishment of a cadre of female community health volunteers (FCHVs) (8). The GoN has prioritized key maternal and child nutrition programs, such as the Nepal Safe Motherhood and Newborn Health Road Map, 2019. In 2011, Nepal joined the Scaling Up Nutrition (SUN) movement that focused on multisectoral and multistakeholder approaches to improving nutrition outcomes, and the GoN then developed its first multi-sectorial nutrition plan (MSNP, 2013–2017) that brought together efforts across health and family planning; agriculture and markets; education; water, sanitation and hygiene (WASH); and other sectors (9). Despite progress between the start of the MSNP and its current third iteration (2023–2027), challenges persist including deeper vertical and horizontal collaboration requiring diverse actors to build relationships and scale-up of implementation of key interventions across sectors at the community level (10, 11).

Nepal’s rapid reductions in child and maternal undernutrition in the past few decades have been documented in multiple studies, including decomposition analyses to identify contributing factors to this success (12). Secondary analyses of national survey data from 1996 to 2016 have also shown continued improvements in some infant and young child feeding (IYCF) practices, such as initiating breastfeeding within the first hour of birth, timely introduction of complementary feeding, child minimum dietary diversity (MDD) and minimum acceptable diet (MAD) (13). Despite this progress, as of 2022, about half of Nepalese children aged 6–23 months (52%) and women aged 15–49 years (44%) had diets that did not meet the MDD requirements (14). Critically, there is stark variation in the prevalence of these ideal practices. Specifically, households from a lower wealth quintile, traditionally excluded caste groups, or living in the *terai* (plains) region often have poorer nutrition practices than their counterparts. For example, in 2022, 39% of children from the lowest wealth quintile versus 67% from the highest wealth quintile consumed diets meeting the standard for MAD. These inequities may stem from lower coverage of programs, limited access to nutritious foods, geographic isolation, and other contributing factors (15).

There is extensive documentation on the benefits of nutrition programs using SBC approaches - interpersonal communication, community mobilization, and mass media (e.g., radio, social media, SMS)—to improve maternal and child nutrition practices (16–18). Greater behavior change success has been seen from programs that combine approaches such as implementing mass media and individual/community education interventions (19) and some evidence suggests benefit from greater intensity of messaging (i.e., reinforcing messages in different platforms) (20).

While the benefits of SBC in nutrition interventions are well supported, there is less evidence on the equity of uptake between more and less marginalized groups and what strategies are needed to ensure that everyone is reached. One study in Nigeria showed that pro-poor interventions could reduce inequities in exclusive breastfeeding (21). Another study in rural India found that frontline workers were less able to reach lower socio-economic status households, and the intentional design of the self-help groups to meet these women with maternal and newborn health interventions would be crucial (22). The common belief that improving intervention quality will inevitably help to close equity gaps has contributed to knowledge gaps on the role of equity in intervention design and measurement (23). In Nepal, inequities go beyond wealth differences and include caste/ethnicity as well as geography. For centuries, one’s caste/ethnicity, assigned at birth in the Hindu system, has determined one’s social identity and social inclusion and directly ties to the opportunities one does or does not have for social mobility. Similarly, given the extreme agro-ecological diversity in Nepal with altitude ranging from 60 to nearly 9,000 meters, geographic inequities persist: those in the *terai* have greater access to roads and markets whereas those in hills and mountains are increasingly remote and isolated. These inequities also interact with each other to create multiple layers of marginalization for some (24).

*Suaahara II*, a USAID-funded program, was implemented in all communities of 42 of Nepal’s 77 districts from 2016 to 2023, building on the first phase of *Suaahara* (2011–2016). This large-scale initiative, aligned with Nepal’s MSNP, aimed to reduce maternal and child undernutrition via interventions primarily in nutrition; health and family planning; WASH; agriculture and markets; and nutrition governance. *Suaahara II* invested heavily in multiple SBC platforms including: (1) interpersonal communication (IPC); (2) community events (CE) bringing groups together for activities such as food demonstrations and key life events (e.g., celebrations of a new pregnancy or a child turning 6 months of age), (3) *Bhanchhin Aama*, a weekly drama initially produced centrally and aired on radio and later locally produced and aired on radio as well as Facebook and YouTube, and (4) SMS messages timed based on age and stage of the 1,000-day period to remind and motivate mothers and family members to seek care and adopt ideal nutrition-related practices. All interventions utilized a gender equality and social inclusion (GESI) approach to target women and disadvantaged groups and focus on GESI-related barriers to adopting promoted practices. Aligned with the complex program, *Suaahara II* had a complex monitoring, evaluation, and research (MER) system, including annual monitoring surveys to assess changes in key indicators over time; MER data was analyzed, and learnings were used to continuously improve intervention approaches and targeting.

The recently published household-level impact evaluation results highlighted that *Suaahara* improved a variety of child health and nutrition practices, but not maternal dietary practices or antenatal care or postnatal care participation (25). Given persistent undernutrition in Nepal and globally, as well as inequities in maternal and child nutrition outcomes, there is a need to study further how SBC interventions can improve maternal and child nutrition behaviors and whether these SBC interventions and their effects are experienced equitably among sub-populations. Using a cross-sectional dataset, our specific research questions include:

1. Is maternal engagement with *Suaahara II* SBC interventions associated with promoted maternal and child nutrition practices?
2. Do the associations between maternal engagement with *Suaahara II* SBC interventions and maternal and child nutrition practices vary by household wealth, caste, or geographic location?

## 2 Methods

**Figure 1:**
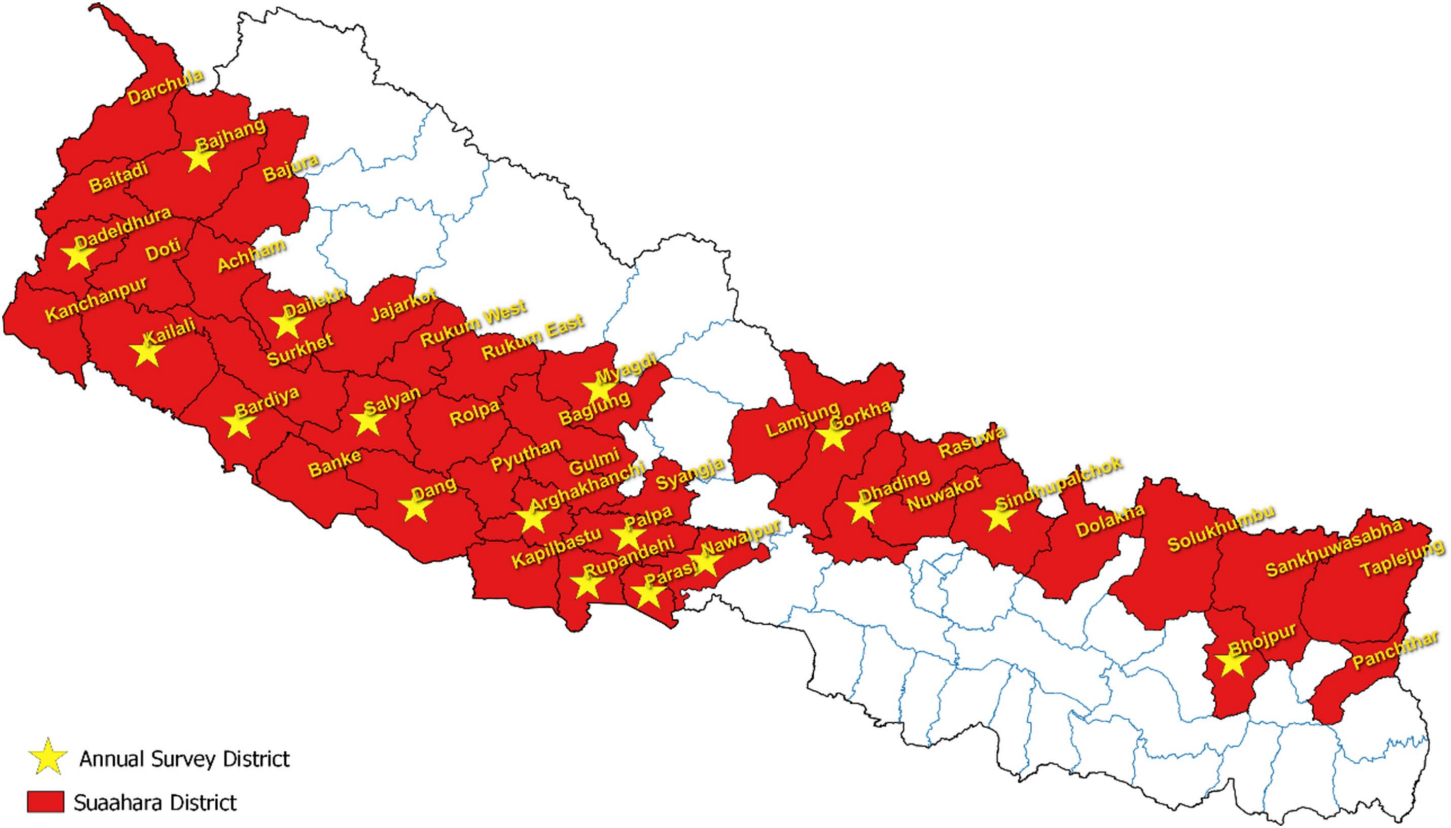
Annual survey districts.

The data used in this study is from the *Suaahara II* 2022 cross-sectional annual monitoring dataset, which was collected by an external survey firm (Figure 1). The survey employed multi-stage cluster sampling at the district, municipality, and community levels, using probability proportional to size (PPS) techniques to randomly select study sites at each level. Ultimately, households with a child aged 0 to 5 years were randomly selected for the study. Primary survey respondents included the mother of the selected child and the head of household (male if available). All analyses are limited to households with a child under 2 years (*n* = 1815) of age given this was *Suaahara II’s* target population and the relevant age group for the child outcomes in this study.

The two primary outcomes were both constructed as continuous variables and included: (1) a scale of 6 maternal nutrition-related practices (antenatal care, iron and folic acid in pregnancy, diet in pregnancy, postnatal care, iron and folic acid postpartum, and maternal dietary diversity) and (2) a scale of 5 child nutrition-related practices (institutional birth, health mothers’ group participation, growth monitoring and promotion, early initiation of breastfeeding and infant and young child feeding—exclusive breastfeeding or timely introduction of complementary foods, depending on child age), with the practices selected based on program focus on these behaviors in the year before the survey, during the COVID-19 context. The primary exposure variable was also constructed as a continuous variable, summing exposure to different platforms to create a scale (0–4) that measures the degree of maternal engagement with the four *Suaahara II* SBC interventions of interest. Three dimensions of equity, identified based on program focus and as key social determinants of health in Nepal—wealth (economic stability), caste (community and social context), and agro-ecological zone of residency (physical environment) - were utilized to answer the final research question, using a binary variable for each.

### 2.1 Outcome 1: scale (0–6) of maternal nutrition-related practices

Antenatal care: whether the mother participated in at least four antenatal care visits during her most recent pregnancy;

Iron and folic acid in pregnancy: whether the mother consumed at least 180 tablets during her most recent pregnancy;

Diet in pregnancy: whether the mother ate more food (i.e., one more meal per day) during pregnancy rather than the same or less than usual;

Postnatal care: whether the mother participated in at least three postnatal care visits after her most recent child delivery;

Iron and folic acid postpartum: whether the mother consumed at least 45 tablets after her most recent child delivery; and

Maternal dietary diversity: whether the mother consumed foods from at least 5 of 10 food groups in the 24 h prior to the survey (26).

### 2.2 Outcome 2: scale (0–5) of child nutrition-related practices

Institutional birth: whether the child was born in a health facility;

Health mothers’ group participation: whether the mother participated at least once in an FCHV-led group meeting on health and nutrition in the 6 months prior to the survey;

Growth monitoring and promotion: whether the child was taken at least once for a growth monitoring and promotion check in the 6 months prior to the survey;

Early initiation of breastfeeding: whether breastfeeding was initiated within 1 hour after the child was born; and

Infant and young child feeding: whether infants were exclusively breastfed, meaning they only received breast milk without any additional liquids or solids, not even water, for the first 6 months (for those 0–5 months) or young children (6–23 months) were introduced to complementary foods (water/liquids, milk other than breast milk, semi-solid foods, solid foods, eggs, and meat) when they were 6–8 months of age.

### 2.3 Exposure: scale (0–4) of *Suaahara* SBC interventions (IPC + CE + BA+SMS)

Interpersonal communication (IPC): whether the mother ever met with a frontline worker in the 6 months prior to the survey;

Community events (CE): whether the mother participated in any community events (i.e., food demonstrations, key life events, etc. led by *Suaahara* staff and/or Female Community Health Volunteers) in the 6 months prior to the survey;

*Bhanchhin Aama* (BA): whether the mother ever listened to *Bhanchhin Aama* prior to the survey; and

SMS: whether the mother received any message on her mobile device about health or nutrition in the month prior to the survey.

### 2.4 Primary dimensions of equity

1. Wealth/Socio-economic status: measuring relative wealth, the equity quintile was generated using asset ownership and materials of the home^1^ to categorize the households as lower 40% versus upper 60%;
2. Caste: categorized with Dalit, Muslim, or disadvantaged Janajati as socially excluded versus Brahmin, Chettri and other non-disadvantaged caste as non-socially excluded; and
3. Agro-ecological zone: residency in the plains (*terai*) versus hills and mountains.

First, descriptive analyses were conducted for socio-economic and demographic variables, outcomes, and exposure variables. Then, linear regression models were run to explore associations between the degree of maternal engagement with *Suaahara II* SBC intervention platforms (dummy variables of 0/1 for each of the four platforms, with 0 being the reference in the models) and both ordinal scales of 6 maternal nutrition-related practices and 5 child nutrition-related practices. Linear regression was done once both outcome scales were confirmed to be symmetrically distributed with the mean, median, and mode in the center of the distribution. Given that the four SBC interventions build on each other and are conceptually challenging to examine as if they are independent along with the vast number of models generated by each platform and each sub-group, we ultimately decided to model with an exposure variable that combines exposure to the four SBC interventions. Lastly, additional models were run separately for each sub-group of the association by wealth, caste, or geography. All models were adjusted for community-level clustering as well as demographic variables that could be potential confounders, including maternal age and education, gender of the household head, household caste, and socio-economic status (measured using equity quintiles) and community-level clustering. All descriptive and regression analyses were conducted using Stata statistical software.

Respondents gave written informed consent to participate in the survey, which was approved by the Nepal Health Research Council in 2019.

## 3 Results

### 3.1 Sample characteristics

Household, child, and maternal characteristics among sampled households are presented in Table 1. Slightly more than one-third of households were headed by a female (about 35%), and slightly more belonged to a socially excluded caste, including Dalit, Muslim, or disadvantaged Janajati (about 53%). A little less than one-third of households resided in the *terai* (approximately 32%) and about one-third (about 34%) belong to the two relatively lowest socio-economic groups/equity quintiles. Children were, on average, 11.9 months old and almost half (about 46%) were female. Mothers were, on average, 25 years old and more than a third of mothers had completed secondary education or beyond (approximately 39%).

**Table 1:** Background characteristics of the study population.

	Year 2022 (*N* = 1815)
	% or mean (SD)
Household characteristics	
Household head sex: female	34.5%
Household caste: socially excluded	53.6%
Household residency: *terai* (vs. lowland plains)	32.0%
Household wealth: lower 40%	33.7%
Child characteristics	
Child sex: female	45.8%
Child age (completed months)	11.9 (6.9)
Child age group	
0–5.9 months	22.7%
6–11.9 months	26.9%
12–17.9 months	22.2%
18–23.9 months	28.2%
Maternal characteristics	
Mother’s age (completed years)	25.3 (5.2)
Mother’s age group	
15–19.9 years	9.8%
20–24.9 years	40.0%
25–29.9 years	30.3%
30 years and above	19.9%
Mother’s education group	
Never attended school/grade 1 not complete	7.2%
Some primary school	8.2%
Completed primary school	7.1%
Some secondary education	38.6%
Completed secondary education	19.5%
Completed class 12/higher education	19.5%

### 3.2 Equity in exposures and outcomes

Overall, about half of mothers were exposed to each of IPC, CE, and *Bhanchhin Aama*, whereas only about 23% of mothers were exposed to the SMS intervention. Engagement with *Suaahara II* SBC interventions differed among sub-populations (Table 2). While more than four-fifths of the sampled population participated in at least one of the platforms, the largest gap found was by agro-ecological zone, with the lowest engagement in the *terai*: about 22% of mothers residing in the *terai* did not engage at all, versus only about 15% residing in the hills/mountains. Similarly, while about one-third (approximately 34%) of mothers residing in the mountains and hills engaged in at least 3 of 4 platforms, the prevalence of this intensity of engagement was less among *terai* mothers (approximately 24%). Maternal engagement with each SBC platform was similar between the two caste groups: engagement with frontline workers was slightly greater among the socially excluded (about 51% versus about 46%) than their counterparts. Among the relatively wealthier, engagement was greater with frontline workers (about 51% versus about 45%), community events (about 55% versus about 48%) and SMS (about 27% versus about 16%) than the relatively poorer 40% of the population. *Bhanchhin Aama* listenership had the greatest geographical difference, with a 21-percentage point gap favoring those residing in the hills and mountains (approximately 61% versus approximately 40%). The geographic difference for mothers having received SMS messages was smaller: 24% of those residing in the hills and mountains versus 21% of those residing in the *terai*.

**Table 2:** Exposure to *Suaahara* interventions and key maternal and child health outcomes by caste, wealth, and geography.

	Total 2022 (*N* = 1815)	Household caste/ethnicity		Household wealth status		Agro-ecological zone	
		Socially excluded (*N* = 973)	Not socially excluded (*N* = 842)	Lower 40% (*N* = 611)	Upper 60% (*N* = 1,204)	*Terai* (*N* = 580)	Hills/mountains (*N* = 1,235)
Exposure to Suaahara interventions							
Interpersonal Communication	48.8%	51.0%	46.2%	44.7%	50.8%	46.0%	50.0%
Community Events	52.5%	53.6%	51.3%	48.5%	54.6%	50.7%	53.4%
*Bhanchhin Aama*	54.4%	54.4%	54.4%	53.2%	55.0%	39.8%	61.2%
Short Message Service messages	23.1%	23.4%	22.8%	16.4%	26.6%	21.2%	24.1%
Scale: none	17.4%	16.3%	18.5%	20.1%	16.0%	22.4%	15.0%
Scale: 1	26.3%	26.4%	26.1%	30.0%	24.4%	27.2%	25.8%
Scale: 2	25.2%	24.5%	26.1%	23.4%	26.2%	26.2%	24.8%
Scale: 3	22.5%	24.2%	20.6%	20.1%	23.7%	18.5%	24.4%
Scale: 4	8.7%	8.6%	8.7%	6.4%	9.8%	5.7%	10.0%
Maternal outcomes							
Antenatal care (4+) during pregnancy	84.9%	85.1%	84.7%	81.8%	86.5%	81.1%	86.7%
Iron and Folic Acid (180+) during pregnancy	65.0%	63.6%	66.5%	61.1%	66.9%	61.2%	66.7%
Eat more during pregnancy	46.5%	46.3%	46.7%	35.7%	51.9%	42.9%	48.1%
Postnatal care (3+)	38.7%	37.3%	40.4%	33.6%	41.4%	34.5%	40.7%
Iron and Folic Acid (45+) during the post-partum period	38.2%	35.8%	41.1%	32.6%	41.1%	37.2%	38.7%
Minimum diet diversity	45.2%	42.5%	48.5%	39.6%	48.1%	35.9%	49.6%
Scale (0–6)	3.18 (1.40)	3.10 (1.34)	3.28 (1.46)	2.84 (1.37)	3.36 (1.38)	2.93 (1.31)	3.30 (1.42)
Child outcomes							
Institutional delivery	87.6%	86.5%	88.8%	81.2%	90.9%	91.6%	85.8%
Health Mothers’ Group participation	28.2%	31.1%	24.8%	24.6%	30.1%	27.1%	28.7%
Growth Monitoring and Promotion participation	85.2%	85.6%	84.8%	87.4%	84.1%	78.6%	88.3%
Early initiation of breastfeeding	66.6%	70.3%	62.2%	68.9%	65.4%	62.9%	68.3%
Exclusive breastfeeding/Introduction of complementary foods (6–8.9 months)	38.7%	39.5%	37.9%	43.2%	36.5%	36.9%	39.6%
Scale (0–5)	3.06 (1.03)	3.13 (1.03)	2.99 (1.03)	3.05 (1.03)	3.07 (1.03)	2.97 (1.09)	3.11 (1.00)

Nearly all mothers (about 85%) had at least four antenatal care visits during pregnancy and nearly two-thirds (approximately 65%) took at least the recommended 180 iron and folic acid tablets during pregnancy. Not even half of mothers (about 38% to about 47%) adopted the other maternal nutrition-related practices (eating more food during pregnancy, at least 3 postnatal care visits, at least 45 iron and folic acid tablets in the postpartum period, and maternal minimum dietary diversity). Some of these practices had at least 5 percentage point equity-related gaps. By caste, the two largest differences were lower prevalence among the socially excluded than their counterparts for taking at least 45 iron and folic acid tablets in the post-partum period (about 36% versus about 41%) and minimum dietary diversity (about 43% versus about 49%). Furthermore, the relatively poorer 40% were lagging in all 6 maternal nutrition-related practices by a range of 5 to 16 percentage points. Similarly, mothers in the *terai* had a lower prevalence by 5 to 14 percentage points of five of the six maternal nutrition-related practices than mothers in the hills and mountains, with the largest gap being for maternal dietary diversity.

Almost all babies (approximately 88%) were born in a health facility, and a similar percentage participated in growth monitoring and promotion at least once in the last 6 months. Not even one-third (about 28%) participated in an FCHV-led health mothers’ group meeting at least once in the last 6 months. Two-thirds (approximately 67%) of infants were breastfed in the first hour of life. Not even half (about 39%) met an ideal feeding standard for their age (exclusively breastfed for the first 6 months of life for those 0–6 months or introduction of complementary feeding between 6 to 8.9 months for those 6 to 23 months). Some of these child nutrition-related practices also had at least 5 percentage point equity-related gaps. A six-percentage point gap was identified for FCHV-led group participation (about 31% versus about 25%) and early initiation of breastfeeding (approximately 70% versus approximately 62%), with higher prevalences among the socially excluded mothers. Relatively poorer mothers had less uptake of institutional delivery (about 81% versus about 91%) and group meetings (about 25% versus about 30%) than relatively wealthier mothers, but the relatively poorer mothers had a higher prevalence of ideal age-appropriate infant and young child feeding than the relatively wealthier mothers (approximately 43% versus approximately 37%). While mothers from the *terai* seemed to give birth more regularly in a health facility (about 92% versus about 86%), the prevalence of their participation in growth monitoring and promotion was lower (about 79% versus about 88%) and the practice of early initiation of breastfeeding (approximately 63% versus approximately 68%) than mothers from the hills and mountains.

### 3.3 Associations between engagement with Suaahara II SBC interventions and maternal and child nutrition outcomes: overall and by sub-population

Maternal engagement with *Suaahara II* SBC interventions was positively associated with the adoption of ideal, promoted maternal nutrition-related practices, with a consistent increase in magnitude of positive association for participation in more interventions (Table 3). Overall, and for all sub-populations other than the wealthy, engagement with at least two platforms was required for a meaningful positive association, and usually, engagement in three or more was needed for achieving one or nearly one (0.70 to 0.97) additional full behavior on the scale of 6 maternal nutrition practices used in this study. In the stratified sub-analyses, effect modification can clearly be seen with variation in the stratum-specific associations on either side of the overall association: the positive associations were slightly stronger for mothers from the non-socially excluded caste, the wealthier, and those residing in the *terai*.

**Table 3:** Associations between engagement with Suaahara II interventions and maternal and child nutrition practices: overall and by caste, wealth, and geography.

	Overall	Caste		Wealth		Agro-ecological zone	
	(*N* = 1815)	Socially excluded (*N* = 973)	Not socially excluded (*N* = 842)	Lower 40% (*N* = 611)	Upper 60% (*N* = 1,204)	Terai	Hills/mountains
Maternal nutrition practices (scale 0–6: antenatal care, iron and folic acid in pregnancy, diet in pregnancy, postnatal care, iron and folic acid postpartum, and maternal dietary diversity)							
0	Reference						
1	0.20 [0.01–0.39]	0.15 [−0.10-0.39]	0.22 [−0.05-0.50]	0.06 [−0.23-0.35]	0.26* [0.01–0.51]	0.46** [0.13–0.80]	0.05 [−0.17-0.27]
2	0.46*** [0.26–0.66]	0.27* [0.01–0.56]	0.67*** [0.39–0.95]	0.48** [0.18–0.79]	0.41** [0.16–0.66]	0.55** [0.19–0.91]	0.37** [0.14–0.60]
3 or 4	0.81*** [0.61–1.01]	0.70*** [0.44–0.97]	0.94*** [0.65–1.23]	0.71*** [0.35–1.06]	0.83*** [0.58–1.07]	0.97*** [0.64–1.30]	0.71*** [0.46–0.97]
Child nutrition practices (scale 0–5: institutional birth, health mothers’ group participation, growth monitoring and promotion, early initiation of breastfeeding and infant and young child feeding—exclusive breastfeeding or introduction of complementary foods)							
0	Reference						
1	0.30*** [0.14–0.46]	0.28** [0.09–0.46]	0.31** [0.09–0.54]	0.20 [−0.04-0.44]	0.34** [0.14–0.55]	0.62*** [0.34–0.90]	0.11 [−0.06-0.29]
2	0.40*** [0.24–0.56]	0.33** [0.13–0.53]	0.47*** [0.25–0.68]	0.21 [−0.05-0.46]	0.50*** [0.31–0.69]	0.66*** [0.42–0.90]	0.24** [0.06–0.42]
3 or 4	0.69*** [0.52–0.85]	0.68*** [0.50–0.87]	0.69*** [0.44–0.94]	0.44** [0.17–0.71]	0.81*** [0.61–1.01]	0.82*** [0.55–1.09]	0.58*** [0.40–0.76]

*p < 0.05; **p < 0.01; ***p < 0.001.

Similar to the results for maternal nutrition practices, maternal engagement with *Suaahara II* SBC interventions was positively associated with the adoption of ideal, promoted child nutrition-related practices, with a consistent increase in the magnitude of association following participation in more interventions. Unlike the finding for the maternal nutrition models, however, overall and for nearly all sub-populations, engagement with even one platform was sufficient for a meaningful positive association to be found. In the stratified sub-analyses, there was little variation by caste. Socio-economic differences in the association were stark, however, highlighting effect modification. While any engagement with *Suaahara II* SBC interventions was positively associated with ideal child nutrition practices among the wealthier, the relatively poor required maternal engagement with at least three platforms for the association to exist. Additionally, the magnitude of association was larger for the wealthier at each level of engagement than for the relatively poorer. Finally, mothers residing in the *terai* seemed to have similar benefits as those residing in the hills and mountains, whether engaging one or two platforms, and only a slightly greater payoff for greater engagement. Effect modification was also found by agro-ecological zone of residency in the stratified models: the overall magnitude of association was much smaller for those in the hills and mountains versus those residing in the *terai*; engagement with even three platforms for mothers in the hills and mountains had a weaker association than for those residing in the *terai* who only engaged with one platform.

## 4 Discussion

In this study, maternal engagement with *Suaahara II* SBC intervention platforms was positively associated with both scales of promoted maternal and child nutrition practices. The degree of association varied by intensity of maternal engagement ranging from 0.2 to 0.8 for maternal nutrition-related practices and 0.3 to 0.7 for child nutrition-related practices. Effect modification was found through the stratified analyses. Wealth variation for both maternal and child outcomes highlighted that engagement with at least two of the four platforms was necessary for the positive association among poorer mothers, versus the positive association found for engagement with one platform among relatively wealthier mothers. Similarly, geographic-based findings suggested the magnitude of associations was stronger for mothers from the *terai* than from the hills and mountains for both the maternal and child outcomes. Models investigating caste/ethnicity differences found less variation but still some: engagement with at least two of the four platforms was necessary for the positive association to be found for the maternal outcomes; the positive association was found even for engagement with only one platform for the child outcomes.

With the most consistent variation found in associations between exposure and key outcomes by agro-ecological zone of residency, these findings suggest that reaching households in the *terai* remains a challenge: population density means that IPC efforts require significant investments in human resources, whereas language variation and a highly mobile population moving back and forth across the border with India present other challenges for program implementation. Another well-documented *terai-*specific challenge is that socio-cultural norms often limit women’s freedom of movement or prevent women’s decision-making about their participation in groups. Creative approaches to overcoming these barriers should be implemented, as SBC efforts appear to have a greater impact on households in lowland plains compared to those in the mountains and hills. Similar evidence for geographic variation in maternal and child nutrition practices but with a focus on urban versus rural have been documented. For instance, a study conducted in India found that urban or rural residence was a predictor in the uptake of infant and young child feeding practices (27). Similarly, another study in Bangladesh found that urban or rural residence was a predictor for skilled birth attendance during delivery (28). Studies looking at other geographic-based variation, such as by agro-ecological zone, were not found.

A review of national datasets and even comparing the findings of these analyses with earlier *Suaahara* publications shows progress over the last decade for many nutrition-related practices (13). The Frongillo et al. paper based on the impact evaluation highlights *Suaahara’s* role in the noted progress for many of these behaviors, particularly those related to infant and young child feeding (26), which is likely not only the effect of direct SBC interventions but the related documented impact of *Suaahara* on the health system and nutrition governance throughout Nepal (29, 30). Ideal maternal and child nutrition practices, however, still require further investment: at least half of the Nepalese 1,000-day population have still not adopted six of the 11 practices in this study. Furthermore, the stratified analyses highlight persistent equity gaps, usually by wealth and geography, but also some by caste. With poorer mothers having lower adoption of nearly all the ideal practices, the GoN and development partners should address barriers for these households that go beyond knowledge and SBC interventions. Similarly, the barriers created for some practices due to remoteness, terrain, or other factors related to agro-ecology must be acknowledged and acted upon to close these geography-related equity gaps.

The finding that maternal exposure to *Suaahara* intervention platforms is associated with ideal promoted nutrition-related practices and that there is a dose–response relationship is encouraging for SBC interventions. These updated data and analyses are consistent with and build on earlier findings of the positive associations, particularly with dietary practices, and the important benefits of more intense exposure (31). The magnitude of the associations found for mothers exposed to one, two, or three/four platforms was similar for the maternal and child outcome scales. It seems, however, that greater engagement is needed for the association with maternal outcomes to materialize, suggesting greater ease of adoption of promoted practices for the child. This is consistent with the main *Suaahara* impact evaluation finding that *Suaahara* did not improve maternal nutrition practices the way it did child nutrition practices, although it did reduce maternal underweight (26). Similarly, the recently published results of the *Suaahara* SMS trial show a positive effect of the intervention on child diets, IYCF knowledge, and other child-related outcomes but not maternal diets among those who received and read the messages (32). There are likely a variety of reasons this trend is emerging, which may be due to greater emphasis on infant and young child feeding than maternal-specific health and nutrition in the implementation of the SBC interventions or also socio-cultural factors, including that caregivers and families tend to prioritize taking care of the child over themselves when resources are tight.

Present-day caste inequity related to nutrition in Nepal is well-documented (33). For the maternal outcomes explored in this study, the positive associations were slightly larger for the upper caste. In contrast, there was almost no difference by caste for associations with child nutrition outcomes. Other studies have also identified that being from a lower caste group in Nepal can act as a barrier to adoption of some nutrition practices, such as early initiation of breastfeeding (34) and ideal complementary feeding practices (35). Since mothers from both caste groups had similar levels of exposure to each *Suaahara* intervention platform, it is likely that there is some socio-cultural barrier(s) among mothers from the socially excluded caste, complicating the uptake of promoted practices for maternal nutritional well-being that do not similarly hinder her uptake of promoted practices for child nutritional well-being.

Wealth-related gaps in nutrition are known (36). These analyses also suggest that Nepal is no exception. Poorer mothers consistently had less program uptake than wealthier mothers; the only platform that reached both at equal proportions was *Bhanchhin Aama.* The fact that the magnitude of associations between maternal exposure to Suaahara SBC interventions and nutrition outcomes was greater for the relatively wealthier households than the poorer households for both maternal and child outcomes also suggests resource barriers that SBC interventions do not address. For instance, service-related outcomes (e.g., antenatal care, postnatal care, growth monitoring) require time to travel and wait for healthcare and sometimes even money to pay for transportation; similarly, dietary diversity and introduction of complementary foods require access to and availability of foods locally and time to prepare foods for a healthy diet. Another study in Nepal found that wealth was a significant factor in explaining variation in the uptake of maternal practices, specifically having a skilled birth attendant present and that access to health promotion activities is inevitably tied to individual, socio-economic, and environmental conditions (37).

Geographic inequities persist for various reasons: distance and road conditions can create obstacles to seeking health and nutrition services, and rural and urban communities have differing access to markets, roads, and services. Additionally, the populations that reside in the lowland plains (*terai*) of Nepal versus the hills and mountains are also distinct with varying cultural practices, migration patterns, languages and more. These analyses show that while three of the four SBC interventions reached equally in the two geographic areas, *Bhanchhin Aama* is not as listened to by mothers in the *terai* as in the hills and mountains. This may be due to a preference for other medium, such as television; less awareness of the program’s existence, or more frequent migration out of Nepal. The finding that the magnitude of association was stronger for *terai* mothers than their counterparts, especially for child nutrition practices, again suggests barriers beyond knowledge particularly for those residing in the hills and mountains. It is likely that the environmental conditions present obstacles to adoption of ideal practices that require dietary diversity and travel to seek health and nutrition services.

From a global perspective, these research findings confirm the importance of having program interventions targeted and tailored to specific populations, with a specific focus on vulnerable households, across different geographic zones, taking into account local contexts and preferences. Likewise, the findings suggest that vulnerable households may need more intense interventions with several components to support behavior change. A similar observation is observed on behavior change in the newly published review by the Board for International Food and Agriculture development on increasing demand for healthy diets (38). The finding that the SMS intervention, which started many years after the IPC, CE and *Bhanchhin Aama* interventions, also highlights that it SBC interventions can take time to saturate target populations. Finally, this study highlights the need to understand local domains of inequity and the possible intersectionality of these domains.

## 5 Conclusions and recommendations

This is one of the few studies to interrogate equity in program exposure and in key nutrition outcomes. Using a large dataset to look across different sub-populations provides insights to guide future programs and policies. However, some limitations should be considered when interpreting the findings. First, these are cross-sectional regression models, so establishing causality is impossible. These analyses have controlled for as many confounding factors as possible. Still, there are likely other factors at play that are not included in our models due to data limitations and being unknown. Also, these analyses looked at each equity dimension separately and did not look at overlapping equity dimensions, which is an important consideration. For example, a mother from the *terai* and a socially excluded caste versus a mother from the *terai* but from an upper caste would fare differently. However, this could not be considered as this would greatly complicate the analyses given the number of potential combinations and in turn, magnify challenges related to statistical power and multiplicity.

As Nepal continues to tackle maternal and child malnutrition, it will be important to keep equity in mind regarding monitoring outcomes by sub-population and designing and implementing interventions so that all segments of the population can engage. Future research studies should ensure they are powered and designed to be able to explore not only the overall effect of interventions but also programmatic effects among sub-populations. Similarly, mixed-methods work will be important to understand the obstacles to intervention reach and uptake of key behaviors among different populations. Additional formative research may also be helpful in enhancing the intervention design itself, to ensure key program interventions are tailored to meet the needs of the communities they serve. While this study contributes to a global evidence base on the importance of intervention intensity, more research is needed to understand exactly how much of each intervention and in what combination and sequence is most effective for effective SBC for all population groups. This type of information would ensure that donors and implementers are well-informed and that investments have the biggest payoff possible for the target populations.

## Data Availability

The data analyzed in this study is subject to the following licenses/restrictions: these datasets have been submitted to USAID. The data used for these analyses are available from the first and corresponding authors, per request. Requests to access these datasets should be directed to Kenda Cunningham, kcunningham@hki.org and Ramesh Adhikari, RPAdhikari@hki.org.

## Footnotes

^1^ https://www.equitytool.org/nepal-2/
